# “It's Your Problem. Deal with It.” Performers' Experiences of Psychological Challenges in Music

**DOI:** 10.3389/fpsyg.2017.02374

**Published:** 2018-01-25

**Authors:** Ellis Pecen, David J. Collins, Áine MacNamara

**Affiliations:** School of Sport and Wellbeing, Institute of Coaching and Performance, University of Central Lancashire, Preston, United Kingdom

**Keywords:** coping, performers, musician, conservatoire, performance anxiety, psychological skills

## Abstract

Musicians need to deal with a range of challenges during their performance career and in response to these have reported a number of conditions that impact on their performance. Although social support from peers and teachers has been identified as part of the process of dealing with these challenges, little is understood about musicians' coping methods, beliefs and their attitudes toward support. Therefore, this study aimed to explore (a) performers' previous experiences of psychological challenges, (b) the types of support they used and, (c) how this might inform future support programs in learning environments. Fifteen interviews were conducted with pre-elite (*n* = 5) transitioning elite (*n* = 3) and established elite performers (*n* = 7) in order to elicit data on psychological challenges, coping, beliefs and preferences for support. Inductive content analysis suggested that elite performers in this sample reported positive health habits, philosophical views of performance, health and life, positive anxiety reappraisal, and use of various psychological strategies, albeit without being explicitly aware of it. The need for various professional skills (e.g., communication, business, self-management, and organizational skills) was emphasized by all participants. Transition into conservatoire was marked by severe psychological challenges, disorders and trauma. Primary sources of support included friends, family and self-help literature. Professional help was predominantly sought for physical problems. The impact of teachers was paramount, yet securing good teachers was considered a matter of “luck.” The most negative aspects recounted included abusive teachers, unsupportive environments, social comparison, competition, and disillusionment after entering the profession. Participants believed that talent could be developed and also valued wellbeing in relation to performance. Positive effects of late specialization on social development and professional skills were also mentioned. Implications and suggestions are discussed.

## Introduction

In order to establish and maintain a successful performance career, musicians need to deal with challenges such as coping with the consequences of early specialization, social isolation, practice volume, identity foreclosure, teacher relationships, burnout, injury, psychological pressure, and perfectionism (Pecen et al., 2016). Other prevalent conditions include psychopathology, musculoskeletal and neuromuscular overuse, irregular work, and sleep schedules (for a review see Kenny and Ackermann, 2009), poor health habits (e.g., Panebianco-Warrens et al., 2014), mood and anxiety-related disorders, and, particularly, music performance anxiety (MPA; for a review, see Kenny, 2011). In response to these health and psychological issues, music students have reported turning to peers and teachers for advice rather than to medical professionals (Williamon and Thompson, 2006). In addition, music learning environments have not yet widely established robust, appropriate, and accessible support for students in the areas of psychological self-management, health, and wellbeing and continued work is needed to promote such endeavors (see e.g., Perkins et al., 2017).

Thus, many challenges need to be overcome and learning environments are likely to be a key location where musicians can be equipped with such skills. However, a number of obstacles still need to be overcome in order to implement appropriate support. One such obstacle might be the limited availability of talent development research in music. For instance, musical talent development has received considerably less attention and implementation than other disciplines such as organizational performance (e.g., Mehdiabadi and Li, 2016) and, particularly, sports performance (e.g., Collins and MacNamara, 2017). As a result, a more detailed and practically-oriented literature base is needed to inform talent development programs in music. Crucially, psychological preparation is an integral part of talent development across performance domains. Traditionally, musical learning environments have focused on selecting “talent” and relying on practice and expert instruction to enable progress. This model, however, does not address the multifaceted aspects of talent development and as a result, does not optimally equip performers for the demands of today's music industry (see Pecen et al., 2016). This is especially relevant as musical success is not solely determined by technical and musical proficiency but also, crucially, by various environmental and psychosocial factors (Subotnik, 2000, 2004; Subotnik et al., 2003, 2016; MacNamara et al., 2008, 2014; Macnamara and Collins, 2009; Subotnik and Knotek, 2009). A positive point is that the same psychological characteristics appear to underpin elite performance across domains and this may hold promise for transferring training solutions from other domains, to music. If cultural and developmental differences would be examined and catered to, positive impact might be maximized and music too might benefit from applied support programs observed in domains such as elite sports. Although there may be cultural and task-related differences, psychologically, there are many convergences. For instance, a set of key psychological skills have been identified in sports (Orlick and Partington, 1988) and have been refined for talent development contexts (e.g., MacNamara et al., 2010a,b) and in music specifically (Talbot-Honeck and Orlick, 1998). Later identified as Psychological Characteristics for Developing Excellence (PCDEs; MacNamara et al., 2010a) such skills include imagery, focus and distraction control, objective performance evaluation and attribution, commitment, planning and organization, goal setting, and self-reinforcement, quality practice, resilience and self-regulation, and, creation and usage of support networks. The additional characteristics of creativity, spontaneity, and flexibility have also been reported in musicians, specifically (Talbot-Honeck and Orlick, 1998).

Reflecting the importance of the aforementioned psychosocial skills as key determinants of success, it is important to examine the coping behaviors and sources of support utilized by performers of different levels of expertise. For instance, given that some research suggests that there may be qualitative differences between pre-elites and expert performers with regards to focus during performance (Buma et al., 2015; Oudejans et al., 2017), it is relevant to examine the coping experiences of this cohort across the performance pathway.

In addressing these issues, research has surveyed and described common challenges in a population of professional musicians (Pecen and Collins, submitted) with the aim of eliciting data on how to construct an appropriate support program. This study affirmed the presence of common challenges (e.g., performance anxiety, injury, mood, and anxiety-related disorders) and also provided specific pointers regarding the content, location, timing and delivery format of a support program. In addition, Pecen and Collins found that musicians used a range of methods to remedy various issues, but often without conveying a clear rationale for doing so. Furthermore, participants seemed to assume that treatments and strategies aimed at increasing experiential wellbeing (e.g., current feelings of relaxation) would automatically lead to enhanced performance. They also appeared to seek help to remedy physical issues rather than prevent them, and seemed less inclined to seek help for anxiety and performance-related problems. The aforementioned survey study highlighted new areas of interest, but it did not allow for an in-depth exploration of the reasons behind why a coping behavior was chosen, why a specific source was sought out or what the detailed context was for the coping behaviors that were used. Thus, while research has repeatedly established the scope of the problem, there is less detailed knowledge of the broader issues contributing toward adherence to certain coping behaviors in musical learning environments. For instance, the impact of thought processes or rationale underpinning chosen behaviors or the influence of cultural beliefs and attitudes toward support. Similarly, the reason for seeking out a particular coping source and how such behaviors might differ depending on where performers are on their developmental pathway. Does their level of expertise seem to have an impact over time, for instance? In short, what is the broader context of a coping behavior that was applied? This is important to inform contextual awareness when using such insight for future practical application in learning environments.

Reflecting these issues and the relevance of addressing the issue in context, this study aimed to explore pre-elite, transitioning elite and elite performers' experienced challenges, employed coping behaviors and sources, impactful beliefs, preferences for support and emergent qualitative differences between groups. A subsequent aim was to use this insight to inspire future support programs in learning environments. Specifically, the research sub-questions explored:

1. The nature and timing of the challenges experienced,
2. What participants did in order to cope,
3. Their beliefs and attitudes,
4. Potentially meaningful differences between subsamples, and
5. Their preferences for support.

## Method

The study aimed to investigate performers' experiences of challenges, coping behaviors, beliefs, preferences for support, and emergent qualitative differences between groups of expertise. The knowledge generated pertained to performers' subjective experiences. The questions were designed to generate in-depth and practically useful data that would help practitioners understand performers' interpretations of their subjective reality within their individual context, rather than to produce a generalizable truth. The ultimate goal of the study was to make use of the insights gained to inspire future practical application in the form support programs. To this end, the study was approached through a pragmatic epistemological lens, accompanied by a qualitative methodology.

Pragmatism focuses on practical solutions and the consequences of the research (Giacobbi et al., 2005) and aims to generate a “practical” truth in a specific context. It acknowledges multiple realities and it seeks to understand the world rather than reveal “the” truth about an objective reality (Giacobbi et al., 2005). Agreement within a community can serve as a “practical” level of truth. To maintain methodological coherence and integrity (Levitt et al., 2017), a pragmatic approach was used to guide the practical intent of the research questions (how to *use* the information we gather?), participant selection (a diverse professional sample) and data analysis (focus on experience and process). Fidelity to the subject and utility to the achievement of goals were maintained by collecting data from various participants, recognizing the influence of investigator bias and limiting this to obtain a clear representation. We also considered the findings in their unique context, collected rich data through methods that enabled meaningful contribution and enhanced utility by comparing the findings against existing knowledge (Levitt et al., 2017).

A qualitative methodology was applied as the study sought to elicit useful results rather than generate an accurate representation of reality (Strean, 1998). Methods were thus selected for their suitability for answering the research questions (Giacobbi et al., 2005). Data were obtained using semi-structured interviews and analyzed using inductive content analysis. This resulted in a “bottom-up” construction of theory where codes and themes emerged from the data a posteriori. These approaches allowed participants to express their experiences of subjective reality and render data that allowed the researcher to explore and understand participants' interpretations of events and interpret how this might be considered for practical effect in the future.

### Participants

Fifteen performers (five pre-elite, three transitioning elite, and seven elite) were purposively recruited via leading UK conservatoires, professional performing arts agents and the first author's personal contacts in the music industry. The sample consisted of seven female and eight male performers aged between 25 and 60 years old, with a clear split apparent between elites and pre-elites (Elite: *M* = 46; *SD* = 7.15, Pre-elite and Transitioning: *M* = 28; *SD* = 3.46). Performers were considered elite if they were currently performing and had achieved expert status in their instrumental disciplines. Typically, the elite performers had established themselves via the conventional route to expert status in classical music. This included: winning major competitions, being signed with leading agents, currently and regularly performing at prestigious venues, being a successful recording artist, and having visiting or ongoing teaching positions at leading conservatoires. Pre-elites were conservatoire graduates who were successfully pursuing a solo performance career that held promise for future elite status. The label “transitioning elite” was given *post-hoc* to pre-elites who differed in that they were on the brink of entering the elite circuit. They particularly distinguished themselves from the other pre-elites on the basis of specific skills and knowledge, details of which will be discussed in the Results section.

Status was verified by the recruitment process through music industry professionals and by reviewing performers' current performance track record and performance engagements. Establishments were contacted to provide a list of successful alumni, from which names were selected and performance profiles were examined. Institutions therefore were not aware of which participants were selected. Finally, to maintain anonymity, data were scanned to remove any clues to participant identity as they were drawn from a tight population. Therefore, demographic details are not included, with participants referred to by letter and number (i.e., Elites —E1 to E7; Transitioning —T1 to T3; Pre-elites PE1 to PE5).

### Procedure

Informed consent was obtained from participants prior to their interviews. A semi-structured interview guide containing open-ended questions, probes, and prompts was developed to allow flexibility to suit performers' individual pathways (Appendix A in Supplementary Material). Questions were informed by current literature and, more specifically, aimed to generate knowledge on those psychological aspects of performance that have thus far received limited attention in research. Prior to interviewing participants, the interview guide was reviewed by the research team and piloted with three pre-elite performers. The ages of participants in the pilot study ranged from 25 to 45 years old (*M* = 32; *SD* = 11). This process resulted in the refinement and clarification of the way some questions were phrased.

**Figure 1:**
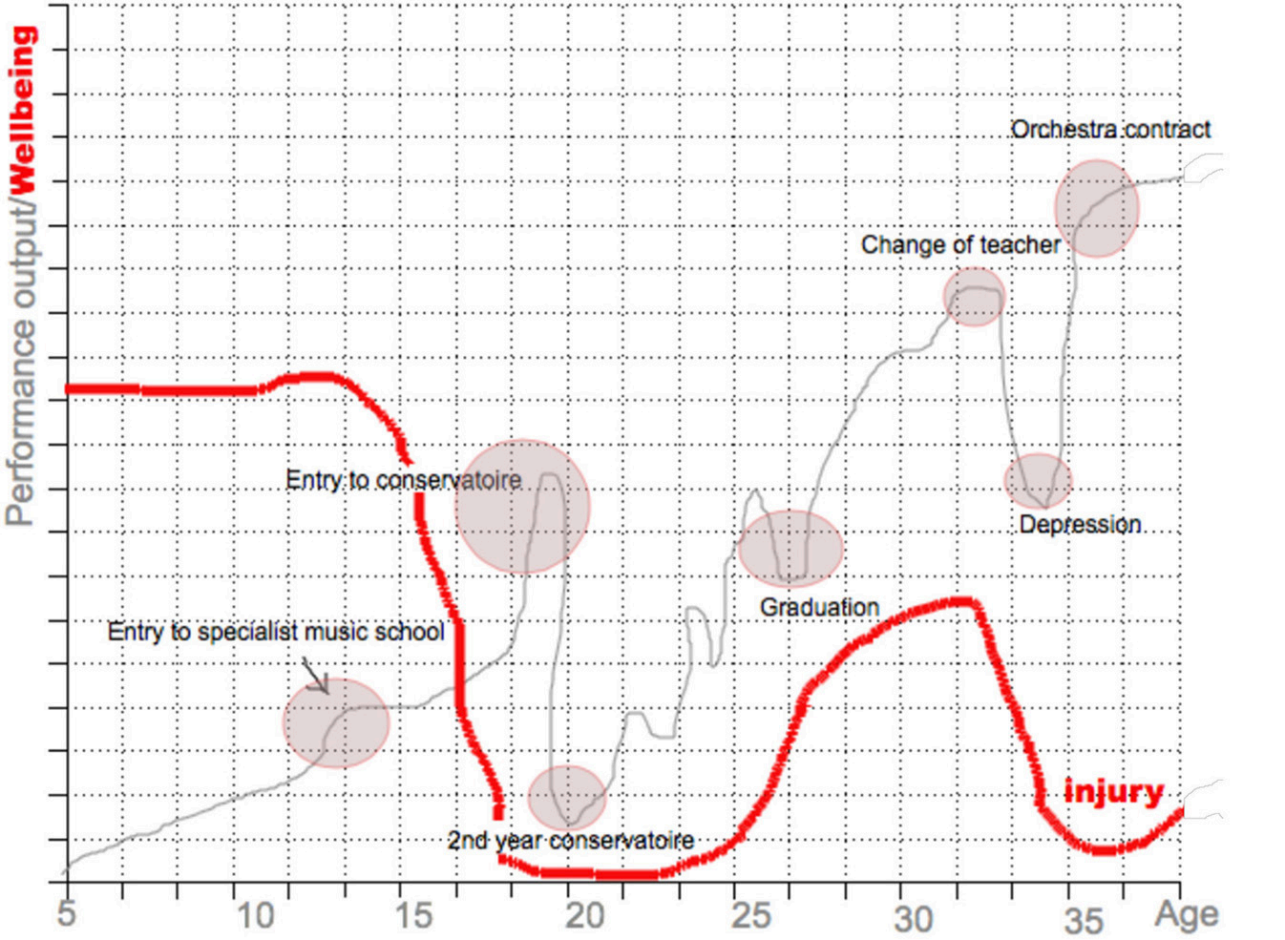
Exemplar copy of hand-drawn graph showing performers' perceived performance output and wellbeing over time (identifying details have been omitted).

To facilitate accurate recall (Drasch and Matthes, 2013), participants were asked to hand-draw a graph of their personal performance pathway (Figure 1). This graph depicted participants' perceived performance over time. Subsequently, participants were asked to mark key events on their pathway (e.g., changes of teacher, phase of injury, entry to conservatoire etc.). Following this, they were asked to plot a second line that represented their personal wellbeing over time, in a different color on the same graph. These timelines and key events were then used as the basis for detailed discussion (cf. Collins et al., 2016). Specifically, participants were asked to comment retrospectively and order their memories chronologically. This method was useful in producing information on key events' and phases' relations to each other and allowed participants to produce critical and interpretative accounts of their past experiences. Interviews were conducted by the first author and lasted between 60 and 75 min (*M* = 68; *SD* = 7.7). Interviews were audio recorded for transcription and analysis and were discarded upon completion of final data analysis.

### Data analysis

Data were analyzed using an inductive content analysis. Stages of analysis included reading through the textual data, coding quotes from participants into units of meaningful text and labeling these, organizing these codes in order to develop descriptive sub-themes by combining similar codes together, connecting and interrelating themes and constructing an overarching, analytical theme that served as the narrative (Creswell, 2002). This process was repeated until codes fitted into five main content categories. After meaning units were identified, these were checked against the purpose of the research question. In the final stage, findings were compared and contrasted to existing literature to assess whether the findings were reasonable (Bengtsson, 2016). In addition, a coding list including explanations of the codes was included in the process to aid reliability by limiting cognitive change during data analysis (Bengtsson, 2016).

### Trustworthiness

Acknowledging contemporary debates concerning rigor in qualitative research (e.g., Smith and McGannon, 2017), we understand the arguments that attempting to establish rigid validity may negate the purpose of qualitative inquiry by imposing positivist norms and that such methods may not necessarily increase validity (e.g., Sandelowski, 1993; Angen, 2000; Smith and McGannon, 2017). Although we cannot assert an independent reality, we have attempted to provide as faithful a representation of performers' realities as possible. This was aided by the specificity of the interview questions and subsequent answers. As such, replies did not require extensive interpretation as the clarity of the questions was reflected by the clarity of the quotes (see Appendix A in Supplementary Material). Considering this, the study sought to establish trustworthiness by adhering to the following procedures in a bid to improve credibility.

Member reflections (Smith and McGannon, 2017) were not practically achievable due to elite performers' touring schedules, and this was a limitation we recognized. Instead, member checks were conducted during the process of data collection, and electronically after data transcription, to ensure that participants' data had been interpreted appropriately. Two participants clarified their answers on two questions at the end of their interviews (during the “in process” member check) yet no further alterations were requested following these steps.

Qualitative data were analyzed using software (QSR NVIVO 11.3.0), which allowed for optimized transparency (cf. Bringer et al., 2004), creation of conceptual memos (Davis and Meyer, 2009), and challenging data interpretation. To remain conscious of interpretative bias, a reflexive journal was kept (Patton, 2002) which allowed for conscious use of the researcher's personal interpretative framework (Levy, 2003). Additionally, a third member of the research team, an experienced qualitative researcher who had not been involved in data collection or analysis, evaluated the transcripts and interpretations of codes against the labels assigned to these by the first author. Following this, results were discussed and triangulated with members of the research team until a structure for the content analysis was agreed upon (Stake, 2008). The second author provided ongoing critique of investigation and interpretations (Faulkner and Sparkes, 1999). Data were subjected to constant comparison in order to evaluate and modify the development of findings (Corbin and Strauss, 2008).

Interviews were conducted by the first author. Process and outcomes were shared by trust and rapport with participants which was aided by the first author's standing as a performing artist and consultant in performing arts, knowledge of the career context and background of the participants and understanding of the cultural milieu. The second and third authors both had extensive experience conducting qualitative research, as well as consulting in elite performance environments including performing arts.

## Results

Reflecting the purposes of the investigation, results from data analysis are presented in four sections as follows; challenges experienced, coping approaches used, preferences for support, relevant beliefs and observed differences between subgroups. Themes and subthemes which emerged through inductive content analysis, are represented in Table 1. Participant numbers (*n* = …) have been included at the end of each subtheme to clarify the total number of participants who made references to the subtheme in question.

**Table 1:** Emerging themes and subthemes following inductive content analysis.

Challenges	Bad teachers (*n* = 15)
	Environmental (parents, discouragement, critique, role modeling, competition, social comparison, late specialization, psychological stigma, institution culture, politics, relocation, travel, adjustment, culture clashes; *n* = 15)
	Professional (changing industry & requirements; *n* = 15)
	Psychological (“crash” around 19 years old, anxiety, procrastination, depression, dealing with criticism, social media negativity, difficulty planning and self-managing, identity foreclosure, personal problems, eating disorders; *n* = 13)
	Physical (injury, appearance, age, energy; *n* = 11)
Coping sources	Good teachers, inspirational role models (*n* = 15)
	Friends & family (*n* = 15)
	Self-help literature (*n* = 10)
	Humor (*n* = 3)
	Preparation (*n* = 15)
	Psychological strategies (*n* = 9)
	Health habits (*n* = 8)
	Substances (drugs, alcohol, alternative medicine, beta-blockers; *n* = 13)
Preferences for support	Characteristics (inspirational, freedom, like-mindedness + preferred early introduction to psychological skills & advice; *n* = 10)
	Complete skills package: Professional (business, networking, media), personality & communication (friendliness, connection, presence), psychological skills; *n* = 13)
Beliefs	Talent & Skill *Development* (*n* = 13)
	Wellbeing & Performance (WB “more important” than/underpinning performance success; *n* = 12)
Differences	Practice: (emphasis on high quantity; pre-elites; *n* = 5)
	Versatility (transitioning elites; self-marketing skills, communication, social media, awareness that conventional career pathway of the established elites is no longer viable; *n* = 3)
	Benefits of challenge (performer persona & coping skills; elites & transitioning elites; *n* = 9)
	Positive health habits (elites; *n* = 5)
	Specific psychological strategies (elites & transitioning elites; *n* = 8)
	Philosophical perspectives (elites; *n* = 5)

### Challenges

The most salient challenges were recounted as being related to bad and abusive teachers, entry into conservatoire, the changing demands of the music industry, unsupportive environments, social comparison and competition, injury, psychological problems, identity foreclosure, work-life balance and personal problems.

#### Bad teachers

All participants (*n* = 15) made passing references to bad teachers and seven participants mentioned having been subjected to very bad teachers themselves. Early tuition was often remembered as lacking in quality until performers were old enough to realize, or were made to realize, that more specialized training was necessary for them to progress. Obtaining good teachers seemed to be regarded as a matter of luck, with seven participants recounting serious problems, such as manipulation, dangerous advice, sexual harassment, and abuse of power in relationships with their teachers.

I felt that my first teacher had brainwashed me to think that if I'm not with him, I will not succeed […]. I felt so trapped. […] When I broke up with my teacher and I wasn't over it and it was more than 2 years, I knew that wasn't normal […] I always needed him more and more and more […] I couldn't do anything without him at all.—PE1.

Three participants recounted being very pleased with their teachers, yet seemed equally aware of their good fortune and how this was not the norm. A teacher's ability to be inspirational and philosophical was particularly valued.

I know so many people who have suffered from teachers who are basically bullies who should not be working with art or with people […]. They are only interested in preserving some sort of school of thought […]. So many people I have met have stopped playing because they weren't put in the right direction or were constantly worked against.—PE2.

Two elite participants had not gone through conventional conservatoire training and expressed an appreciation for their broader knowledge base and having been given more freedom outside of institution culture. Despite this, perceived performance output and wellbeing increased for all participants after leaving conservatoire or university education.

There were no cons other than having fewer contacts to arrange ensembles with. It was all pros, absolutely. Because I self-guided and was curious and motivated, I read a wide variety of things, there were no restrictions.—E7.

#### Environmental

Environmental influences impacted participants' experiences of challenge and all participants (*n* = 15) made references to its impact. For example, discouraging and competitive environments were referred to frequently by the participants of all subgroups (*n* = 13). In addition to the previously mentioned references to specific teachers, critique was also continuously voiced of the larger educational establishment and institutional culture that was also viewed as often working against students (*n* = 13).

But the music school, that was almost cruel. Two weeks before my auditions for senior college they told me that “I shouldn't do them because I wouldn't get into any of them anyway”… and that's a huge knock to anybody. Support should have been in place there […]. It's an educational institution. Surely it's their job to make you achieve as much as you can achieve and make you feel as good as you can about what your capabilities are. And literally, they did the opposite […]. They chisel you down to nothing and then, they're like “BYE!”—T1.

Competition was viewed as a means to an end, as an artificial situation that did not suit the purpose of music and that was riddled with biases and politics (*n* = 12).

I am not a fan at all of competitions […]. The first and second winners are often students of the jury […]. You are playing for a very fake and artificial situation.—E1.

Three performers mentioned being berated for late specialization and how this had detrimental effects on their psychological wellbeing. In contrast, these participants described the benefits of their late specialization and how it had a favorable impact on their social development and the subsequent development of a “complete performer skills package” or even ability to remember how to explicitly explain skill development.

It was just so hard. All the time I felt that I was being reminded by everybody that I was never going to make it. You know “you'll never make it, why don't you do this or this instead?”[…]. You're too old, you started too late' […] but, I had a great childhood. I had no practice, no pressure […]. I'm actually so happy that I didn't start at the age of four because then practice becomes life. It would have changed my personality.—T1.

A lot of people who start with a good teacher at five years old, they don't remember […]. But in my case there are a lot of things I can explain because at ten years old I had to learn like somebody who had an accident and having to learn how to use their legs or something. Because of that experience I think I have knowledge I wouldn't have if I'd been like those people.—E3.

Frequent references (*n* = 14) were made to stigma of psychological problems, “keeping up appearances” and a perceived environmental requirement that dictated a need to “hide problems,” which was considered common in the domain. However, four participants expressed disagreement with this and suggested that:

It's probably not as forthcoming as other industries maybe and other environments. It's a bit hush. It's like they want to pass it on quickly like nothing's happened you know. I don't know what the reason is for this, but it's not good.—T1.

Almost all participants (*n* = 13) recounted having traveled to other countries in order to study. Clashes of cultural differences, moving away from home and stress related to relocating were recounted as key features of these transitions.

My teacher in Russia would disagree with the school of playing in Asia and then in London other teachers would say other things. So you had to play according to how their “School” wanted you to play.—PE3.

Another participant remembers how having to move to an affluent area impacted their body image and self-esteem.

Everybody around looked so perfect. It was a big mental shock that effected my playing and eating. I just didn't want to play anymore and didn't have the energy. I was so tired all the time. I was so unhappy.—T1.

#### Professional

All participants (*n* = 15) drew attention to how the music industry's demands had changed and how this had profoundly impacted their lives on several levels. Various skills such as networking and “public relations” were deemed necessary for a successful and sustainable career and the traditional career path was no longer perceived as relevant.

The music business has changed so much over the past 10 years. You used to have to be very good at playing to be signed to the major classical labels. Now you just need to wear a nice dress and look marketable. Back in the days you won competitions, got an agent, a manager etcetera. Now musicians are having to do so much of these things themselves […]—E1.

[…] The tendency is that the music world has turned into complete 100% business. […]. Too much YouTube, too much free stuff […]. The more sex appeal you have the more audiences you will win and as much as they are actually talented players, I'm absolutely against it. For me music is a world on its own, you don't have to sell it, at all […]—E3.

In response to this, the need for a complete performance skills package was emphasized (*n* = 13). This included communication, self-marketing, stage presence, organizational skills and effective use of social media—skills which appeared to have been learnt autonomously.

I didn't realize it when I was studying because everything was just about technique, but after graduation and trying to find concerts as an artist, I realized how important it was to be like “the complete package.” And no one told you look good, be nice, communicate well, sell tickets, be someone who just intrigues people'.—PE3.

#### Psychological

A noteworthy finding was that almost all participants (*n* = 13) recounted having gone through a traumatic psychological “crash” around the age of 19 years old, ~1 year into their conservatoire training. In contrast, entry into specialist music college (±age 13) was recounted as mostly positive (*n* = 6). Common performance-related problems during the initial years of conservatoire training included isolation, comparing oneself to peers, competitive environments, and performance anxiety. Problems of a more clinical nature included depression, panic disorder, eating disorders, thoughts of suicide, and addiction to drugs and alcohol. Three participants mentioned extreme dieting and carbohydrate restriction for aesthetic reasons.

In my second year I had a very deep depression. Which I had never had ever. I didn't play at all, […]. I was obsessive dieting. I was going to the gym everyday and only eating 600 calories a day […] I wasn't coping.—T1.

Another participant recounted a mixture of clinical problems and a teacher's response:

I really lost it about a year into college. I was starting to hyperventilate, I couldn't sleep anymore and I even became suicidal after a while. I told one of my teachers and he said it was normal.—T3.

Three participants pointed out how the effect of emotion in music may have exacerbated their emotional problems, due to musicians being required by the profession to be overemotional at times, possibly making them more vulnerable.

Musicians are definitely screwed. But it's only because our profession, just like actors, is forcing us to experience life in a much more sharp emotion than normal people. The teacher is telling us “play more musical, feel more this, play with more emotion.” everything is just enlarged […] So I think that is why we are so sensitive.—E3.

Various psychological skills for developing excellence (PCDEs; MacNamara et al., 2010a) were reported by all participants (*n* = 15), yet some were not. Those that were rarely reported as being used effectively, included goal-setting, coping with pressure, planning and organization skills, quality practice and realistic performance evaluations. Various other psychological problems were recounted but the most salient were difficulties dealing with anxiety, depression, stress related to self-management, job insecurity and fear of memory lapses during performance.

I still get so nervous every time I play but the memory lapses, they're the worst. I can just forget everything I am doing, no matter how well I know the piece. Memory is such a source of worry for me. More than technique probably.—PE3.

The impact of isolation and developmental issues was mentioned in the context of social development and identity (*n* = 8). Some performers wondered whether development of music skills was at the cost of developing other life skills that might have benefitted their career.

If you can't be a top performer, then who are you? It's like I don't know anything else other than music and I want to live life but my mind just doesn't know how to do anything else. I try to, but practice just keeps in the back of my mind.—E6.

I had a normal free childhood. I played […]. I'm actually so happy now that I didn't start at the age of four because then practice becomes life. It would have changed my personality. So I'm pleased.—T1.

Most participants (*n* = 13) also suggested that personal problems (e.g., relationship breakdowns, homesickness, family issues) impacted on their performance. This was often attributed to loss of focus, motivation and energy, and decreased ability to focus on performance preparation and execution.

When my boyfriend broke up with me, that was the only time my performance really went down because I just wasn't practicing anymore for a long time, I just didn't know what to do with my life anymore.—PE4.

I ran away from home, I did a lot of teenage stuff. […] My dip was more to do with life than performance. It was a very strict system, you had to justify everything you did to the rector assistant, it was like being at school. Not really performance-related stuff, you just had to go through the system.—E4.

A major source of psychological worry that was often reported (*n* = 14) came from comparing oneself to peers on technical, professional and socio-economical grounds.

I came from a disadvantaged background and suddenly I was in the middle of all these players who had famous teachers. I would never catch up. Mentally, that was the worst bit.—T3.

I'm often very frustrated with feeling like I'm always a second follow-up on everyone's list of everything and that sort of confidence lack. And I think some of my friends and wife etcetera what they did during college, great […]. Because then I could also rely on things like I'm not useless, I got a good mark so I'm good, you know. But now there is no such thing, you see.—PE2.

#### Physical

Accounts of physical problems (e.g., injury, playing-related pain) were common (*n* = 11) due to practice load, volume, and suboptimal lifestyle habits. While participants seemed considerably more prone to seek help for physical issues, references to stigma of injury were also made (*n* = 14).

I started struggling with my back and shoulder early on. Alexander Technique and foam rolling helped but it's still keeping me from practicing. You don't want people to know that you have an injury so I don't go to doctors and have people think I am broken.—PE4.

Also, concerns over body image and impact of physique were described by 13 participants. Exercise was often used in order to stay in shape for performance demands but also for aesthetic reasons (*n* = 8).

We have to increasingly, in an image driven age, keep fit […]. So I have to lose some weight […] But stuff like that is very important because people hear what they see. […]. If I want work that artistically means something, it means that any way that I can get the audience coming back and paying those tickets is a useful thing.—E7.

### Coping sources

Sources of coping included good teachers, friends and family, self-help literature, preparation, health habits, psychological strategies, and use of substances.

#### Good teachers

Inspirational, knowledgeable, open-minded, philosophical, and supportive teachers were mentioned as having a positive impact on coping (*n* = 13). In addition, participants who recounted having had good teachers from their early development years onwards (*n* = 8), seemed to have developed a more robust “psychological buffer” to performance anxiety.

I had a very good relationship with my teacher. He was like a grandfather […]. He made sure I never started to think of the stage as something I feared. I get nervous, but I think it's more excited. I love the buzz and I know it will go well because of that.—PE5.

#### Friends & family

One of the most prominent sources for coping were friends and family (*n* = 15). Professional help seemed to be sought out mostly for physical problems and to a much lesser extent for psychological issues, with only three participants mentioning therapy.

I did the preparation and got through things. I wasn't actually enjoying it. I did reach a point where I seriously considered packing up and leaving in my second year. Sometimes friends and family keep you going, when you are going through a rough patch. A bit of medication did help if I'm brutally honest.—E2.

In addition, positive role modeling by parents and others was mentioned by five participants as having had an impact on their psychological resilience and work ethic.

I always saw my parents be active and work. They would go to work if they were ill […]. I think this mentality of work with this thing that I can do whatever I want helped.—PE2.

#### Self-help

Many participants (*n* = 13) placed an emphasis on self-reliance in terms of coping with the challenges of the domain and displayed a belief in, and preference toward, experiential learning.

You have to rely on yourself because it has to come from within you, that's true talent I think. Help from outside is sort of “nice” but it's not “real” help if you know what I mean? You just have to fix the problem on your own and others can maybe take your mind off things for a bit to make you feel a bit better for a while, but in the end, it's all up to you.—E6.

Six participants recounted using self-help books to overcome challenges. They mentioned ease of access, avoidance of psychological stigma, general enrichment of knowledge, and its applicability to performance. Three participants had sought out therapeutic counseling and also referred to how self-help books were relevant to solve performance problems. Two participants also considered their problems “perhaps not severe enough to justify a therapist's time” and therefore turned to self-help.

I was so isolated that I couldn't communicate well with therapists. Or maybe they were just really bad, I don't know. I felt like my problems weren't “bad” enough and they acted like I was wasting their time with this performance stuff and they were actually music college therapists. But self-help really helped me to deal with performance stuff. […] I read a lot of books about positive thinking and doing things you are afraid to do.—T3.

#### Humor

Three participants referred to using humor as a coping strategy.

Humor is a coping skill for me 100%, always has been. I think in everything I do in life, whether I walk on stage for a concert or do a talk, a meeting with big investors, multimillionaires, it relaxes everybody and gets them on your side.—T1.

I like humor because I like people to be relaxed in company. I don't want people to feel uptight. I think people remember things when you're funny. I just think there are too many miserable bastards in the world who take themselves far too seriously, especially in the musical world and often without justification […]—E2.

#### Preparation

All participants made references to the importance of preparation (*n* = 15). However, practice *planning* seemed mostly absent and preparation seemed to increase in quantity as performances neared. Only three participants mentioned having a plan in order to help them cope with practice, scheduling, performance, and other commitments. In addition, participants, especially pre-elite, seemed to prioritize quantity of practice as the most important factor in their preparation, with anything <1.5 h being considered “not worth it.”

There's nothing you can do in less than 3 hours, just because you have to get warmed up. […]. I have this system whereby I split up the pieces, I learn a little bit every day. I have a blackboard and I have all the pieces and all the pages and how many sections they have and I write down what I need to do for each. I go “do I remember the first section of this piece? Do I remember the second section of that piece?” So you keep track of how much of each piece you know.—E7.

A more worrying finding was that confidence was primarily, and often exclusively, viewed as a the consequence of (over)preparation (*n* = 13), which seemed to encourage overtraining and also showed how participants had a limited view of what constitutes confidence and the various ways in which it can be used to performers' benefit.

You can't feel confident unless you are 200% prepared. So you need to prepare so much that you can justify feeling confident. It's the result of practice.—P5.

It is actually the preparation that deals with all the problems. I don't know why people get so nervous.—E1.

I used to think that I never practiced enough and so I was never confident enough. Almost I was thinking like “I could have not slept and practiced instead” maybe then I could feel more confident. But that just never happened. I sort of just had to act confident and make the best of it and actually I started improving technically after that attitude. I never felt confident from practice.—E6.

#### Psychological strategies

The use of psychological coping skills seemed more pronounced in the transitioning and elite samples (*n* = 9), yet even amongst them, identifying strategies as “psychological” seemed difficult. This was also reflected in the manner in which participants answered these questions. For instance, they seemed to be having difficulties distinguishing between musical, technical and psychological skills, often identifying only musical and technical skills. Continued probing and explanation were often necessary on part of the interviewer to identify psychological skills. Psychological skills also appeared to be used mostly unconsciously with little declarative knowledge of why or how they were used and they did not seem deliberately planned for, practized or refined in a systematic manner. However, six participants (two pre-elite, one transitioning elite, and three elite) were able to describe some psychological strategies they used following probing. Yet even they often seemed unaware that some of these were in fact “strategies.” Even when using techniques that might be considered quite “advanced” in the literature. For instance, one elite described a planned alternation between conscious and subconscious processing using self-talk, performance and memorization cues.

You have to program yourself in a very conscious way, by using the part of your brain –[…] You have to actually use words to put instructions into that area of your brain. Maybe a sort of road sign or cue. Like “now is the passage that begins in E major so you have to…” So that you get through the structure of the piece and you have an actual conscious approach […] and at times the autopilot can take off and you're at one with everything.—E5.

Various psychological characteristics for developing excellence (PCDEs; MacNamara et al., 2010a) were used to cope. Commitment, focus, and self-awareness were the most commonly reported by the participants (*n* = 15). Six participants specifically mentioned the importance of focus and emotional regulation.

First of all, enormous concentration and being able to concentrate on whatever it is very important. The enormous level of control over your emotions. This we need for an actual performance as well. At the same time, the combination of being able to at the same time control yourself and express yourself. To be able to do different things.—T2.

You need to have good nerves. Because if you are a very nervous person this will be pretty hellish. You have to have a cool brain and a hot heart. Because if you get too cold on stage it's not good.—E4.

I did visualization exercises. Imagine your life, a perfect day, or your life in five years' time, the things you'd like to be doing. Things like that. Career planning I guess.—PE1.

#### Health habits

Elite players (*n* = 6), in particular, made references to positive health habits for coping with anxiety, preparing for performance and maintaining a good physique. Two elite participants referred to consuming simple and complex carbohydrates pre-performance as well as the importance of exercise and sleep as part of their performance preparation.

Don't numb yourself, you have to be present. Sugary things that give you energy can help actually. I watch my health habits very much and stay fit. That helps a lot.—E5.

I have wrestled with depressive things throughout my life quite significantly, […] I started [exercising] which I find helps my physical fitness and mental state […]. If I'm in good physical shape I can cope better. And the usual things, I like to eat well, control when and what you eat before you play, and what you wear.—E5.

I've had to learn the hard way. I never used to exercise because of this and that and waste of practice time and things. But then things get out of hand. I started gaining weight, my endurance was shot, I didn't sleep well then I started exercising because I HAD to because I was in so much stress. Then things got better and I also started eating well before a performance.—E6.

#### Substances

Views on the use of substances to cope varied amongst the participants but substances were repeatedly referred to (*n* = 13). Ten participants mentioned that the use of alcohol and drugs in relation to performance was a maladaptive coping characteristic and these were easily identified by all participants as commonly observed bad coping strategies. Five participants mentioned a history of using alcohol or drugs themselves to cope with performance pressures, yet in hindsight acknowledged they did not consider it a positive coping behavior unless there were underlying medical issues. Using drugs and alcohol for the purposes of performance “enhancement” was mostly shunned (*n* = 10). This was mentioned with regards to technical and psychological proficiency, but also the meaning ascribed to being a genuine artist.

Betablockers and that stuff. Absolutely horrible idea […]. I haven't tried it and I wouldn't. Because people are there to see you. You owe it to them. You have to be a genuine artist.—E4.

What I really don't like is alcohol and drugs, that's just fleeing away from fear and not fighting it. Don't do that, feel the music.—E5.

In contrast, three participants mentioned a more lenient stance toward substances and changing their minds about drugs after seeing role models use drugs.

My attitude is democratic. If you are doing a fantastic job as a player I don't care if you are drinking or drugging […]. I know someone if he doesn't have his drugs he's a disaster on stage, but after drugs he's amazing. So as far as I'm concerned, if his character comes out best when he is on drugs, that's his problem.—E4.

I remember I used to have a very judgemental attitude towards drugs and alcohol but then I entered the music world and I sort of saw all these amazing people do it. And the thing is, a lot of them are actually really clever people. My attitude changed from thinking it was just something for druggies to that it's something even clever people do, so I became more okay with it to be honest.—E6.

### Preferences for support

#### Characteristics

Valued characteristics of interventions and providers of support included: having inspirational teachers and speakers who showed open-mindedness, had a sense of perspective and could see the bigger picture, were philosophical, and would give artists freedom to express themselves (*n* = 10). With regards to interventions, participants were mostly in favor of them, saying learning environments shared a responsibility and that introduction to such initiatives ought to happen as early as possible when commencing musical training (*n* = 12).

Sometimes you can have an inspirational teacher who can tell you one thing and it can completely change your way of thinking. I think education has a role to play certainly like that. And I think they have a responsibility to say “stick with it, you can do it.”—E2.

#### “Complete” skills package

As mentioned earlier, challenges related to the changing needs of the industry (e.g., increased emphasis on looks, the traditional pathway to success no longer being relevant, increased reliance on self-marketing etc.) and the subsequent necessity for performers to possess a varied professional skill set (e.g., business, marketing, communication, networking, social media skills) caused many to express a need for more holistic interventions that addressed psychological and health-related aspects, as well as the organizational and business-related aspects of professional life. The majority of participants (*n* = 13) referred to the importance of having skills other than only musical skill in order to address the varying demands on the contemporary music industry.

The ideal thing is probably something that teaches you what's good for your body and your mind. But I think this marketing thing I keep mentioning, […] college as a whole will not really think like that so much […] you would need a whole course for this understanding of what it means to actually BE a musician. The sad thing is that playing is such a small part of it.—PE2.

Some participants (*n* = 10) also emphasized the importance of having a kind, likable personality as well as strong communicative skills.

I have learnt the importance of a nice personality. No matter how well I played I lost out on opportunities because producers didn't want to work with me anymore. You really have to work on having a nice personality to everybody who sees you.—E3.

Psychological skills that were most commonly reported as being vital to learn and teach were perseverance, resilience, commitment and focus (*n* = 13).

The ability to concentrate on music and ideas you need to deliver is the most important […] concentrate on the music, on what the composer says, his ideas, not on your anxiety, or the technical difficulty […] move your mind from this stuff to the real music.—T2.

### Beliefs

#### Talent & skill development

Thirteen participants expressed that they believed talent was mostly developed over time. They also showed a belief in a growth mindset, often saying that achievements were due to commitment and hard work and that talent alone was of limited use. The overarching belief of “the classical music environment” was seen as contrasting participants' views of talent, with 10 participants referring to the domain's “fixed” views on talent and talent selection.

It's still the thing that they expect the right ones will be selected by the right people to do the right thing. But that might not always be the case.”—PE2.

I'd say most of it is actually practice. I used to believe it was talent. You can have some sort of affinity or something but that's no guarantee at all. You can't tell that to the schools and panels of course. I suppose there needs to be something there but probably 20% talent 80% work.—T3.

#### Wellbeing & performance

A noteworthy finding was the importance ascribed to feelings of subjective wellbeing in relation to performance (*n* = 12). The graphs participants drew of their pathway showed that performance output curves and “wellbeing” curves were approximately matching over time (see Figure 1). It seemed wellbeing was prioritized over performance output and/or that evaluations of performance success were heavily influenced by emotional wellbeing. 11 participants also expressed that they felt wellbeing was a precursor to performance success.

If I went on stage and performed the best performance I ever gave in my life and I wasn't feeling right, I wouldn't become happy. It would be like, great… another paycheque.—T1.

In contrast, four participants mentioned they managed to keep performance output high despite feeling low on wellbeing.

I remember I had a dip because of something at home with one of my parents who got ill suddenly. That did affect my wellbeing, but not my performance really. I kept playing. I think I used playing to distract myself. I don't know how well I played though but I was playing. You probably need to feel good to play your best though.—PE5.

My personal life never really affected my performance because I think it's a co-existing parallel. Your artist world is your artist world and you never stop thinking about it. There were moments when due to personal problems and personal cataclysms, from one hand you find it very difficult to perform physically and emotionally, but from the other hand this performing is the only thing that can save you from suicide or something very tragic, because of stupid things that happen to you in life […].—E5.

### Differences across levels of expertise

Whilst pre-elite, transitioning elite and elite performers identified a number of commonalities, some noteworthy differences were observed across these groups. This was reflected in how frequently certain themes emerged among a specific player group or were absent from another. Although these results are not generalizable, it is worthy of pointing out these differences as they did highlight certain tendencies linked to professional attitudes and generational differences.

#### Practice

In the context of practice habits, all but one pre-elite (*n* = 5) still appeared to be striving toward accumulating high quantities of practice. In contrast, the transitioning (*n* = 3) and elite performers (*n* = 6) typically recounted having limited time to practice because of other professional commitments. In addition, they also had discovered how to maintain performance quality without engaging in high practice quantity.

If you think logically, you can't possibly be perfection. Just by deduction, so therefore you are doing wrong things. Now of course it is tricky to know what those wrong things are. But if you keep repeating them, you're going to make those wrong things potentially worse. So I think variety is very important, so you don't do the wrong things for too long. So I think in a very unscientific way, variety and not too much length of time and also it should always be broken up. Never 3 hours.—E2.

This was in contrast to young pre-elite graduates (*n* = 6) who were more likely to report striving toward high quantity practice, as was modeled in their recent education.

Practice, practice, practice. If you feel insecure, you just haven't practiced hard enough. Like, now I only do five hours per day since I graduated, that's the minimum. There's no other way if you want to be good, you just have to.—PE2.

So all this practicing they teach you, it's like, I now need to teach myself not to think that way anymore or I go crazy, but there's still that idea that somehow you have to practice all the time. If I did what I thought was the “right” thing, I would practice all the time.—PE3.

#### Versatility

Transitioning elites (*n* = 3) were particularly versatile, communicative, knowledgeable of the changes in the industry and had found a way of self-managing and adapting to its demands successfully. They were also active in multiple and other areas of performance-related endeavors (e.g., documentaries, research, motivational speaking). Elites (*n* = 6) had gained similar skills, although seemed to need it to a lesser extent professionally as they had established themselves via the more conventional pathway to success and often had staff who helped take care of admin and PR. Although pre-elites (*n* = 6) *mentioned the need* for such skills, they appeared to be less proactively engaged in versatile career activities and only made passing references.

It's not all about the playing and the music. We're performers and we have fan bases and that's what it's about. It's about cultivating your brand on the basis of what you do and who you are. You can be the best performer in the world but you can't string a sentence together because you are too introverted and you can't do interviews, so you can't do it.—T1.

While no participants refuted the importance of versatility, the pre-elites (*n* = 6) particularly did not seem to emphasize the importance of pursuing broader opportunities and skills and seemed more entrenched in prioritizing practice quantity. As a result they seemed disillusioned over how career success did not result from practice hours clocked.

I just had a breakdown in the summer. I'm just doing all this practicing and there are just no gigs. It's like “why did I study this stuff?” I'm just practicing but I don't have concerts basically. Like where to start to get even noticed?—PE4.

#### Benefits of challenge

In addition, transitioning elite (*n* = 3) and established elites (*n* = 6) showed more positive views of anxiety, labeling it as “excitement” or something they didn't allocate their attention to.

I use the stress to get me focused and fired up. I read through a list I have that I write all the stuff that matters to me on before I go onstage—E6.

I think sometimes music conservatories are rather bad about focusing on what's NOT perfect and what needs improving all the time, so you get this terribly negative way of evaluating how things went. Getting it in proportion is very important […]. You have to make a virtue of the extra tension because it is a hell.—E4.

For me the ability to concentrate on music and the ideas you need to deliver is the most important thing […]. Concentrate on music, on what the composer says, on his ideas, not on your anxiety, or the technical difficulty […] move your mind from this stuff to the real music.—T2.

Whereas, in the pre-elite category, only one of five expressed similar views and seemed to be more pre-occupied with worries over the effects of anxiety.

I just get so nervous. And then the lapses start. My hands will be freezing cold and sweaty. And all I can think of is just “don't mess up” and “what's mom going to think?” It's just so hard not to think of anything else when your body's like that.—PE3.

Similarly, the notion of performers potentially benefitting from challenges and trauma, was more prevalent amongst transitioning elite and elite performers (*n* = 9). This was particularly highlighted in relation to developing an “interesting artistic personality,” experience the realities of life and strengthen coping abilities.

It's especially wrong to be overprotective of children. They need to be hardened. Where did this attitude come from? Why do they this is right?—T2.

It sort of feels good because you know you are so deep into it, you feel it so much [the trauma], you need that to become someone who entrances the audience. In a way it also makes you feel like you tried as hard as you could […]. So I think the pain and setbacks have sort of made me more used to dealing with things and know what I need to work on. Also all the personal problems I think made me tolerate stress a lot better […]. I became really good later on in my career and those other people actually sort of settled down or one even quit […].—E6.

#### Positive health habits

In addition, health habits and exercise were more frequently recounted amongst transitioning and elite performers (*n* = 9) and to a much lesser extent by pre-elite (*n* = 6), of whom only one referred to unplanned low impact exercise in order to relax.

I did yoga and I used to go walking and running by myself, walk around feeling grateful.—PE1.

If you don't keep seriously keep fit, you're just going to get fat in this profession, just sitting down all the time practicing.—E3.

I don't exercise or anything. I don't have time and I'm also afraid to make injuries worse because I don't know how.—PE3.

I went to physiotherapists and osteopaths. They told to swim and exercise and sometimes the time is little but what I really do because it saved my life once before, I have an elastic band and I do 15 exercises I know.—E1.

#### Specific psychological strategies

Across participant categories, the most noticeable and advanced psychological strategies were employed by one pre-elite, two transitioning elites and five established elites, who seemed more apt at describing their awareness and the specific strategies they used.

I really make sure that I visualize everything I do in practice in detail. I will do it by section and characteristics and I also combine it with lines in my head. Like, I will use specific words and instructions. Never leave it to chance. Same for memorization.—E4.

In contrast, the reporting of psychological strategies seemed less common in the pre-elite category with only one out of seven participants describing strategies in detail, often resulting in vague accounts. In addition, performers' replies suggested that they struggled with distinguishing between psychological and technical or musical skills. For example, when asked to suggest a specific psychological skill they used, participants often replied by describing technical or musical skills and needed (sometimes persistent) probing in order to arrive at a psychological one.

You just have to perform and just keep doing it until you get used to it. Or you just have bad memory […]. It's a mystery, I don't know.—PE5.

You have to think about how you stand and hold your arm and making your movements small and efficient […]. The music really has to speak the way you had planned.—PE2.

#### Philosophical perspectives

Another noticeable characteristic that distinguished pre-elites from the transitioning elites and established elites was the frequency of their philosophical views (*n* = 9). Established elites seem to view music from a broader perspective, often drawing analogies with other fields and philosophy (*n* = 8).

When you discover what reality is made of, it's a very powerful tool for players. But it's deeply connected to the consciousness of your own being which is neverending […]. When I was young I was an athlete […] I remember very clearly that before the start you listen very clearly for the start shot and then you don't focus on anything else you hear after that […] you are in trance […]. This was also a good experience for my brain because there were similarities with music performance on stage. […] I think only a few artists try to do this authentic deep thing, the other are just players. If you meet the top ones, you would be astonished about how their thinking is.—E1.

This was absent in the pre-elite and to some degree also the transitioning elites, as the emphasis in the latter category seemed to be more on understanding and catering to the demands of the commercial music industry.

Some people will try to tell you all this deep stuff but really it's just a craft plain and simple. Who is your audience, what's your skill and how are you going to sell your brand? That's all it is in reality.—T3.

A lot of people are going to want to come across as these massively profound people […] they say “oh you take the phrase and you have to shape it.” No. As my teacher used to say it: No. Just use the metronome […]. The point of music training is: hey, play! I don't even care if you're sick or you're not feeling good or your cat died or something […]. It is in a sense an athletic act.—E5.

## Discussion

This study aimed to explore pre-elite, transitioning elite, and elite performers' experienced challenges, employed coping behaviors and sources, impactful beliefs, preferences for support and emergent qualitative differences between groups. A subsequent aim was to use this insight to inspire future support programs in learning environments.

### Timing and nature of challenges

This study confirmed and elaborated the findings of previous research in conservatoires and talent development in music (e.g., Subotnik et al., 2016; Perkins et al., 2017) about the timing and nature of challenge. For example, the incidence of psychological and physical problems, power of the teacher, and competitive learning environments that have been previously established (for a review, see Pecen et al., 2016; Perkins et al., 2017) were further evidenced in the current study. Major challenges included relocation, bad and abusive teachers, social comparison, competition and a severe “psychological crash” at around 19 years old were identified. This finding mirrored, but also amplified the findings of Hildebrandt et al. (2012), who found increased levels of fatigue, depression, and stage fright in first year conservatoire students. While research has explored transitions in the development of musical pathways (e.g., MacNamara et al., 2008), the severity and frequency of events recounted in this study are important findings, especially as these are the accounts of the successful few who have negotiated such transitions effectively. It seems pertinent therefore that practitioners and institutions consider preparing students psychologically *in advance* of the critical transition to conservatoire as well as providing accessible performer-focused psychological support *during* this stage. Learning how to use psychological skills and how to express this in words would be beneficial to a performers' own performance, as well as their teaching ability.

Concerns over body image were frequently mentioned, with three performers having previously engaged in extremely restrictive diets. This finding resonates with Kapsetaki and Easmon (2017) who found a high incidence of eating disorders amongst musicians attending conservatoires. Although eating disorders are also common in sports, caloric requirements, and macronutrient ratios between carbohydrates, proteins, and fats, are typically tracked and planned so a particular physique can be obtained without compromising performance. In music, little attention is given to such planning, even though there are accounts of high stress lifestyles, cardiovascular, and stamina demands (see Pecen et al., 2016). Thus, performers are left to their own devices and may engage in dangerous behaviors to achieve the body they think is required by the industry. This can be counterproductive as high energy expenditure in combination with overrestriction of calories or inappropriate macronutrient ratios may lead to e.g., fatigue, stress, and compensatory eating responses (Horswill et al., 1990; Costill and Hargreaves, 1992; King et al., 2007; Tomiyama et al., 2010). It seems prudent to investigate this in more detail, avoid one-size-fits-all dietary advice and consider the performer in context, to counter a potentially vicious cycle between stress and dieting.

### Coping

Goal-setting, coping with pressure, objective performance evaluation and attribution, planning, and organization skills, quality practice and realistic performance evaluations were often lacking in the reports of the participants. These findings are consistent with existing research in music performance that has reported a lack or absence of planning and goal-setting behaviors (e.g., Talbot-Honeck, 1994; Hatfield, 2016). It might be no coincidence that some of these skills are closely related to the business and self-management skills, which were also reported as missing. With regards to goal setting, it also became clear that a personal definition of what constitutes “success” was needed along with subsequent acquisition of skills to teach performers “how to achieve *their* idea of success.” Practitioners might consider how to cater to the subjectivity of career success in the twenty-first century, along with tailored provision of the necessary organizational and psychosocial skills to achieve it.

### Beliefs and attitudes

Participants ascribed a great deal of importance to wellbeing and appeared to consider it a precursor to successful performance. While the idea of “wellbeing equals or leads to good performance” may seem intuitively appealing, this view should be explored further from a performance perspective. It is possible that these perceptions were due to a retrospective evaluation bias. For example, they *felt* bad during or after the performance and therefore *remember* their performance output as bad; they judged their performance on the basis of how they were feeling (for a review, see Kahneman, 2011). However, it is also possible that due to the emotional nature of music and the meaning ascribed to music making, musicians may *want* to “feel good” whilst performing or just “enjoy” the performance. This was also reflected in participants' views of competitions, which were considered a means to an end (Hays, 2002) and were often recounted as artificial, political and not in harmony with the purpose of music making. This might, for instance, contrast with performance psychology in sport. Athletes might train to reach an emotionally uncomfortable level of arousal that they know will benefit their performance outcome. If enduring discomfort leads to a medal, that's a price worth paying. Whereas enduring discomfort for a subjective goal associated with emotion, such as music, may not resonate with musicians if the ascribed goal is enjoyment. Additionally, as it is difficult for musicians to quantify performance output, perhaps they are more likely to turn toward wellbeing for evaluating performance, as “feeling good whilst performing” might be their main means of evaluation. The overarching theme of *aspiring* toward wellbeing during music performance, should be considered when tailoring interventions. Is it possible to “feel good” whilst experiencing the fight-or-flight response? How to incorporate this goal when humans are only capable of experiencing one emotion at a time? It seems reappraisal or reconditioning of cognitive, emotional and behavioral responses to fear-related symptoms, altering perceptions or working toward abandoning this aspiration may be aspects worthy of consideration.

### Emerging differences among elite, per-elites, and transitioning elites

Differences were most commonly observed across the areas of health habits, perceptions of anxiety, philosophical perspectives, and possessing the “complete skills package.” In line with research on deliberate practice (Ericsson, 2006), elite and transitioning elite reported more positive health habits for increased wellbeing and performance, made more references to maximizing practice quality over quantity, and believed that overcoming trauma would be beneficial for coping skills and the development of a resilient personality. Elite players showed a tendency to apply a broader, philosophical perspective. They showed reappraisal of anxiety, viewing anxiety as positive and seemed able to divert their attention away from negative symptoms. Such shifting of focus and viewing of the “bigger picture,” might be a particularly useful area to work on with performers, especially given the emphasis they placed on versatility. Working on focusing on the bigger picture of life as well as performance-related issues, might aid coping by providing perspective, but also serve as an opportunity to practice applying a different type of focus, which could also be used in a performance context. For instance, learning how to shift focus from “narrow” and “internal” to “broad” and “external,” depending on need, is a key psychological skill (Nideffer and Sagal, 2006).

### Preferences for support

Preferences for support in this sample were in line with previous research (e.g., Hays, 2002) yet also emphasized the importance of inspiration, open-mindedness and versatility in presentation and content. Participants suggested that an introduction to such initiatives should happen as early as possible in musical training. Practitioners and specialist music schools might, for instance, consider how to tailor psychological strategies to make them accessible to children.

Three performers pointed out how therapy had failed them as performers (including therapy offered in conservatoires). In this regard, practitioners and institutions might consider the possibility of recruiting professionals who have an understanding of performers and their specific needs. This is important, as performers who are extremely invested, may likely be a performance-oriented, goal-focused, and driven population who want psychological performance solutions as well as increased wellbeing. Some might even exclusively view wellbeing as a consequence of performance improvement. Practitioners must be sensitive to this “performer mindset” and identity, as interventions and approaches that are based on the needs of the “average population” or with, exclusively, the goal of “wellbeing” in mind, may fail. For instance, behaviors may be present that are facilitative in a performance environment, yet that might be deemed pathological by normal standards (cf. Lebrun and Collins, 2017).

Overall, participants were mostly focused on problems regarding the changing demands of the profession and how a varied skill set was vital. This included not only technical and musical proficiency, but also psychosocial, business and communication skills. While this has already been pointed out in scholarly literature (e.g., Bennett, 2016) the *psychological* impact of performers' adjustments to the contemporary industry was a novel addition. The contrast between their anticipated career prospects, their training and the reality of the profession gave rise to psychological struggles and problems with self-identity, organization and time-management. Addressing these issues does not necessarily require overnight systemic change of the conservatoire model. Practitioners and teachers might for instance draw attention to the reality that awaits students, encourage uptake of extra-curricular activities, or even free online training courses (e.g., MOOCs), as self-help was also considered a valuable resource in this study. Especially catering to the individual's pathway based on what they consider to be “future success” seems pivotal, as different definitions of success would require an emphasis on different skills.

## Conclusion

The findings of this study suggest that interventions for musicians might take into account how to train musicians in a number of psychosocial skills, how to prepare them psychologically for key transitions, how to deal with competition and social comparison, how to allow them to be challenged and deploy those skills in the real world. Detailed guidelines are beyond the scope of this paper. However, in order to comprehensively address the wide-ranging challenges outlined, we would suggest an approach akin to the PCDE approach (MacNamara et al., 2010a,b). The important feature of this is that, rather than focusing on a particular skill to address a particular challenge, e.g., resilience (Sarkar and Fletcher, 2014) or Grit (e.g., Duckworth et al., 2007), it provides the performer with a comprehensive toolbox of skills from which he/she can select and deploy to meet the specific challenge at a particular time. It also offers a greater range of empirically validated tools in *addition to* focus, imagery, goal-setting, arousal regulation and self-talk (see MacNamara et al., 2010a). For example, one highly relevant items that is sometimes overlooked in psychological skills training is “knowing how to seek, create and use social support.” And, finally, the messenger matters. A bespoke blend of *performance* psychology specialists, inspirational speakers and self-help methods might be considered to circumvent the stigma of psychological interventions and encourage take-up.

### Limitations

Although not considered a true limitation in qualitative research, for the purposes of constructing future interventions, the small sample (*n* = 15) and the possibility of findings being altered by self-preservation and retrospective recall bias, need to be considered and findings of this study should not be generalized (cf. Arkin et al., 1980). As every method has its pros and cons, interviews rely on interaction, verbalization, conceptualization and memory (Mason, 2002), which may be influenced by self-presentational bias, specific motives, and the impact of dynamics of power relationships between researcher and participants (Charmaz, 2006).

In addition, participants interviewed in this study were all performing at a high standard and had successfully progressed on their individual pathways. They did not succumb to performance pressures and consequently drop out, for instance. We therefore need to consider that the views expressed by these participants are the accounts of “the successful few” who are likely to have acquired the requisite psychological skills and insight into performance and hence are subject to “survivor bias.”

Also, many of the performers who responded to the invitation had favorable views of performance psychology or showed noticeable analytical and philosophical insight. Participants were, as a result, receptive, enthusiastic, open to discussing these issues, thoughtful and aware of themselves and the demands on performance. It is therefore likely that this study has painted an overly positive picture, just as an investigation into MPA may overemphasize the negative aspects of anxiety.

However, it is worth noting the severity and frequency of the challenges participants described. This exemplifies that even highly successful performers have had to overcome considerable adversity in order to reach their current status. It is irrefutable that many musicians do suffer and fail to successfully negotiate the challenges on their performance pathway. The results of this study should therefore be interpreted with care, just as with other retrospective accounts, yet can be used to inform our thinking about how applied research might address such issues sensitively.

These problems notwithstanding, however, it is positive to see the recognition and importance accorded to positive and proactive coping by these performers. Especially considering the severity of events recounted. Further work might track through transitions to conservatoire, considering the perceptions of those who fail as well as those who succeed and work to address the areas of communication and psychological skills, with perhaps a particular consideration for addressing wellbeing in relation to performance, planning, evaluation, and organizational skills, and inspirational, performance-specific support, and self-help methods.

## Ethics statement

Ethical approval was granted by the University of Central Lancashire Research Ethics Committee for Business, Arts, Humanities, and Social Science (BAHSS 444).

## Author contributions

EP was primarily responsible for data collection, analysis and interpretation. Contributions to conception design were made by DC. DC and ÁM provided ongoing critique, review, and researcher triangulation. DC and ÁM have provided final approval of the submitted manuscript.

### Conflict of interest statement

The authors declare that the research was conducted in the absence of any commercial or financial relationships that could be construed as a potential conflict of interest.
