# Supplementary material for: “It's Your Problem. Deal with It.” Performers' Experiences of Psychological Challenges in Music
Source: Front Psychol. 2018 Jan 25;8:2374. doi: 10.3389/fpsyg.2017.02374 (PMC5788962; doi:10.3389/fpsyg.2017.02374)
Supplement: Supplementary file 1 [file DataSheet1.docx]

**Appendix A: Interview Questions**

1. *To begin, I would like to get to know more about you and your musical pathway. Could you briefly introduce yourself?*
   - How old are you?
   - Have you had to relocate for music?
   - Do you live alone? (Support)
   - What instrument do you play?
   - For how long have you been playing this instrument?
   - At what age did you begin?
   - What is your typical practice to performance load in an average week?

*2. What does ‘coping’ with performance pressures mean to you?*

- Could you give me some examples of good and bad coping strategies?
- What makes them good or bad?

*3. Using the timeline of your career, can you give me up to four examples of psychological challenges you have experienced and how you dealt with them?*

- What were these challenges like? Can you briefly describe them?

1. *How common do you think that the challenges you’ve experienced are in your performance environment?*

- Do you know peers who have experienced similar issues?
- What is the attitude of the environment towards such challenges?
- Is talking about it ok or not?

*4. How would you describe the supportiveness of your environment?*

- Who helped you in dealing with the issues you’ve described earlier? What did this support look like?
- How would you describe the ‘attitude’ of your performance environment towards support?
- Is help available?
- How do you know where or who to turn to for the appropriate advice?

5. *Do you believe that some skills can be taught and learnt in music in order to deal with those challenges? If so, where and how did you gain these?*

- Are good performers ‘born’ or do people just have ‘talent’ to cope better and be successful?
- Where or from whom did you learn the psychological skills you use?
- How do think about learning environments equipping students with psychological skills?
